# SEEMLIS: a flexible semi-automated method for enrichment of methylated DNA from low-input samples

**DOI:** 10.1186/s13148-022-01252-4

**Published:** 2022-03-10

**Authors:** Tamara S. Rodems, Duane S. Juang, Charlotte N. Stahlfeld, Cole S. Gilsdorf, Tim E. G. Krueger, Erika Heninger, Shuang G. Zhao, Jamie M. Sperger, David J. Beebe, Michael C. Haffner, Joshua M. Lang

**Affiliations:** 1 University of Wisconsin Carbone Cancer Center, Madison, 1111 Highland Ave., Madison, WI 53705 USA; 2 Department of Pathology, University of Wisconsin, Madison, 1111 Highland Ave., Madison, WI 53705 USA; 3 Department of Medicine, University of Wisconsin, Madison, 1111 Highland Ave., Madison, WI 53705 USA; 4 Department of Human Oncology, University of Wisconsin, Madison, 1111 Highland Ave., Madison, WI 53705 USA; 5 Divisions of Human Biology and Clinical Research, Fred Hutchinson Cancer Research Center, 1100 Fairview Ave, N., Seattle, WA 98109 USA; 6 Department of Pathology, University of Washington, 1959 NE Pacific St., Seattle, WA 98195 USA; 7 Department of Pathology, Johns Hopkins School of Medicine, 600 N Wolfe St., Baltimore, MD 21287 USA; 8 Present Address: 7151 WI Institutes for Medical Research, 1111 Highland Ave., Madison, WI 53705 USA

**Keywords:** DNA methylation, Low-input, Rare analyte, Semi-automation, Circulating tumor cells

## Abstract

**Background:**

DNA methylation alterations have emerged as hallmarks of cancer and have been proposed as screening, prognostic, and predictive biomarkers. Traditional approaches for methylation analysis have relied on bisulfite conversion of DNA, which can damage DNA and is not suitable for targeted gene analysis in low-input samples. Here, we have adapted methyl-CpG-binding domain protein 2 (MBD2)-based DNA enrichment for use on a semi-automated exclusion-based sample preparation (ESP) platform for robust and scalable enrichment of methylated DNA from low-input samples, called SEEMLIS.

**Results:**

We show that combining methylation-sensitive enzyme digestion with ESP-based MBD2 enrichment allows for single gene analysis with high sensitivity for *GSTP1* in highly impure, heterogenous samples. We also show that ESP-based MBD2 enrichment coupled with targeted pre-amplification allows for analysis of multiple genes with sensitivities approaching the single cell level in pure samples for *GSTP1* and *RASSF1* and sensitivity down to 14 cells for these genes in highly impure samples. Finally, we demonstrate the potential clinical utility of SEEMLIS by successful detection of methylated gene signatures in circulating tumor cells (CTCs) from patients with prostate cancer with varying CTC number and sample purity.

**Conclusions:**

SEEMLIS is a robust assay for targeted DNA methylation analysis in low-input samples, with flexibility at multiple steps. We demonstrate the feasibility of this assay to analyze DNA methylation in prostate cancer cells using CTCs from patients with prostate cancer as a real-world example of a low-input analyte of clinical importance. In summary, this novel assay provides a platform for determining methylation signatures in rare cell populations with broad implications for research as well as clinical applications.

**Supplementary Information:**

The online version contains supplementary material available at 10.1186/s13148-022-01252-4.

## Background

Epigenetic modifications to DNA are fundamental to human biology, including histone tail modifications, changes in chromatin structure, and DNA methylation. The ability of epigenetic modifications to alter gene expression without changing the sequence of the genome is essential to human development and disease [1]. DNA methylation in particular has been widely studied for its contribution to biological development and the initiation and progression of various diseases [2]. DNA methylation refers to the addition of a methyl group to the fifth carbon of the nucleotide cytosine. In humans, cytosine methylation mainly occurs at cytosines that are 5′ to a guanine, termed CpG [3]. CpG methylation contributes to gene regulation directly by blocking binding sites of transcription factors or RNA polymerase and indirectly by recruiting other epigenetic modifiers that promote chromatin reorganization [3–5]. Importantly, these changes are heritable and conserved, but can also be plastic in nature [6, 7], making them attractive targets for studying disease progression and developing biomarkers and therapies.

DNA methylation changes are a hallmark of almost all malignancies [8]. In prostate cancer for instance, DNA methylation alterations have been extensively studied and have been shown to exhibit exquisite biomarker properties for early detection and disease monitoring [8–13]. This is particularly relevant since there is a lack of recurrent genomic alterations in prostate cancer [14], with the most common genomic alterations only occurring in ~ 50% of patients [15–18]. This generates a need for non-genomic prognostic and predictive biomarkers. During tumor progression, specific gene promoters and CpG islands are hypermethylated, leading to silencing of genes including tumor-suppressor genes like *APC* and *RASSF1* [19–21]. Importantly, many of these genes are methylated in more than 75% of patients, with some genes like *GSTP1* being methylated in over 95% of patients with prostate cancer [19]. Therefore, DNA methylation changes, especially in specific gene promoters, have the potential to be powerful prognostic and predictive biomarkers.

An increasing number of recent studies have investigated the feasibility of using methylation signatures in DNA from liquid biopsies including circulating tumor cell (CTC) DNA and cell-free DNA (cfDNA) as prognostic and predictive biomarkers. Studies in prostate cancer cfDNA and breast cancer cfDNA and CTCs, for example, have identified multiple candidate biomarkers that may be useful for early detection and diagnosis [22–25]. However, robustly assessing DNA methylation changes from small amounts of DNA remains a major technical challenge. The overwhelming majority of these studies utilize bisulfite conversion-based approaches like methylation-specific PCR (MS-PCR) or reduced representation bisulfite sequencing (RRBS). Bisulfite conversion has been shown to extensively damage DNA and can result in a loss of up to 90% of DNA yield [26]. Due to the limited amount of tumor DNA in liquid biopsies, bisulfite conversion-based approaches are suboptimal and may not enable clinical diagnostic use.

To circumvent the issues that arise from bisulfite-based approaches, multiple studies have investigated affinity enrichment-based approaches using either antibodies or proteins that specifically bind methylated DNA. For instance, the use of a 5-methylcytosine (5mC) or 5-hydroxymethylcytosine (5hmC) antibody has been shown to be able to successfully enrich for methylated DNA from as little as 0.5 ng of starting material [27, 28]. The methyl-CpG binding domain (MBD) of methyl-CpG binding proteins such as MeCP2 and MBD2 have also been used to enrich for methylated DNA [29–31]. An assay termed combination of methylated-DNA precipitation and methylation-sensitive restriction enzymes (COMPARE-MS) employs the methyl-CpG binding domain of MBD2 (MBD2-MBD) coupled with DNA digestion with methylation-sensitive restriction enzymes to enrich for methylated DNA in heterogenous samples. This assay was used to profile multiple genes from 20 ng of DNA from prostate cancer biopsy samples [31]. Here we sought to develop a robust and scalable enrichment platform for targeted analysis of methylated DNA from low-input samples. We combined the strength of the COMPARE-MS assay with Semi-automated Exclusion-based sample preparation (ESP) to Enrich for Methylated DNA from Low-Input Samples, in an assay called SEEMLIS. We developed SEEMLIS to enable methylated DNA analysis in low-input samples such as CTC-derived DNA.

Our laboratory previously developed a well-established platform for CTC capture, protein staining, and nucleic acid extraction both with a handheld and with a semi-automated microfluidic ESP system [32–34]. Both of these systems harness the rapid, gentle, and low-loss physical characteristics of the ESP system to enable extraction of high-quality DNA from low-input samples, which can be used for virtually any DNA-based experimental endpoint. In this study, we show that DNA extracted from this system can be used directly for DNA methylation analysis by SEEMLIS. We validated the performance of SEEMLIS for single gene analysis using CTCs from patients with prostate cancer as our source of low-input target DNA. Additionally, we show that SEEMLIS followed by a pre-amplification step can allow methylation analysis of multiple gene targets from low cell inputs, with sensitivity down to a single cell for certain targets in samples with low non-tumor cell contamination. Finally, we show the potential for SEEMLIS to be used to detect multiple gene targets in CTCs with additional clean-up by single cell aspiration.

SEEMLIS was designed for seamless integration into our CTC capture, imaging, and nucleic acid extraction ESP-based system. However, this assay is applicable for use with any genomic DNA source where methylation marks and genomic integrity are sufficiently preserved. The clinical utility of this assay is far reaching, including the ability to monitor responses to epigenetic therapies and to identify patients with specific methylation signatures that are prognostic of disease outcomes or render them resistant or susceptible to certain therapies. Importantly, while we have developed this system in the context of prostate cancer, this assay is readily transferable to other types of cancer and non-neoplastic diseases where DNA methylation changes are of importance.

## Results

### Range of detection of GSTP1 promoter in LNCaP DNA after digestion with a methylation-sensitive restriction enzyme and MBD2-MBD enrichment

**Fig. 1:**
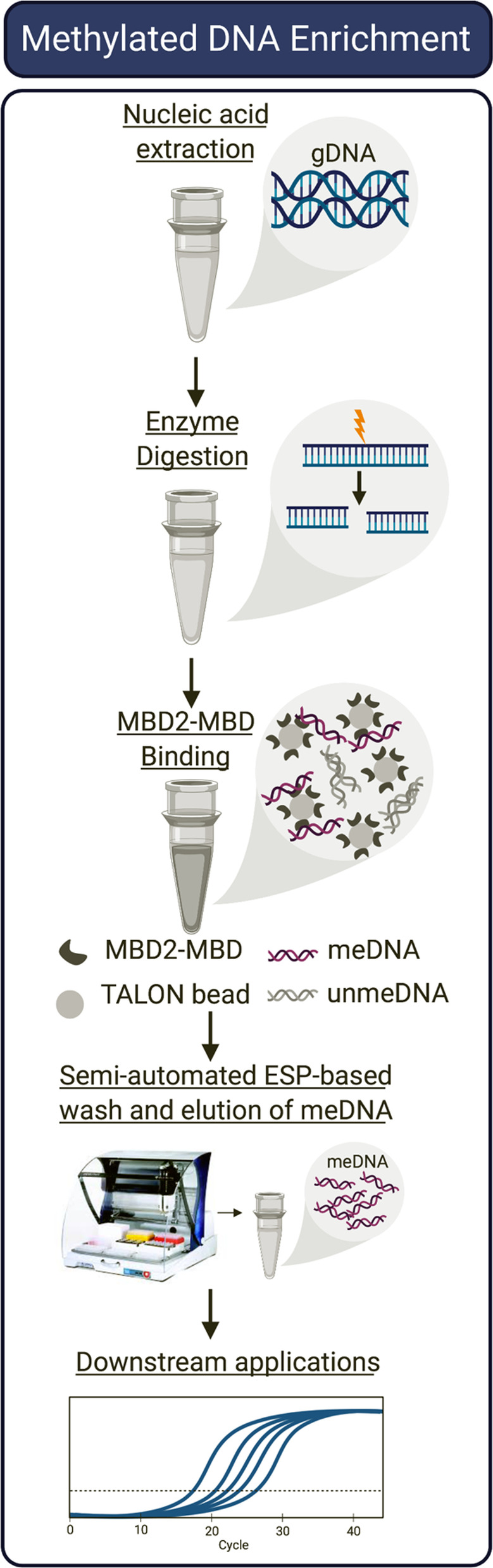
Workflow of SEEMLIS assay. Genomic DNA is digested with restriction enzymes and enriched using MBD2-MBD-bound magnetic beads. Methylated DNA is washed and eluted on a semi-automated ESP-based system and can be used in downstream applications such as qPCR, sequencing, and high-throughput analysis

An overview of the SEEMLIS workflow is shown in Fig. 1. To perform MBD2-MBD enrichment, DNA can be isolated from samples of interest in any way that preserves methylation and does not significantly damage or shear DNA [31]. In this study, DNA was extracted from lysed cells by magnetic silica-coated beads using ESP-based approaches, which our laboratory has used extensively for nucleic acid isolation [32, 34, 35]. To fragment DNA, a combination of methylation-sensitive and methylation-insensitive restriction enzymes were used as described previously [31]. The addition of a methylation-sensitive restriction enzyme that only cuts if the restriction site is unmethylated further reduces unspecific background signal. Tagged MBD2-MBD protein was immobilized on cobalt-coated magnetic beads to capture methylated DNA. Bead-bound DNA is purified by automated magnetic transfer via ESP through a wash buffer and into water for elution.

To test the range of detection for a single, methylated gene, we chose *GSTP1* as a benchmark. *GSTP1* promoter hypermethylation is present in more than 95% of prostate cancers and has previously been used in various biomarker studies [12, 19, 36, 37]. Methylation of the repetitive element, *LINE1*, was used as a positive control for successful enrichment of methylated DNA, since *LINE1* is highly abundant in the human genome and known to be methylated in virtually all human cells [38, 39]. We chose to use the LNCaP cell line as our positive sample. Bisulfite sequencing has previously been performed on the LNCaP cell line, which shows that the promoter and CpG island of *GSTP1* are heavily methylated [31, 40, 41]. White blood cells (WBCs) from healthy donors and patients with prostate cancer were used to represent the WBC population in circulation as our negative control. It has been previously shown that *GSTP1* is hypomethylated in white blood cells [19, 42]. *GSTP1* primer location, genomic context, and methylation levels for LNCaP [41, 43] and WBCs [42, 43] are shown in Additional file 1: Figure S1. For all LNCaP and WBC validation experiments, DNA was extracted by semi-automated ESP as described in the methods unless otherwise indicated.

**Fig. 2:**
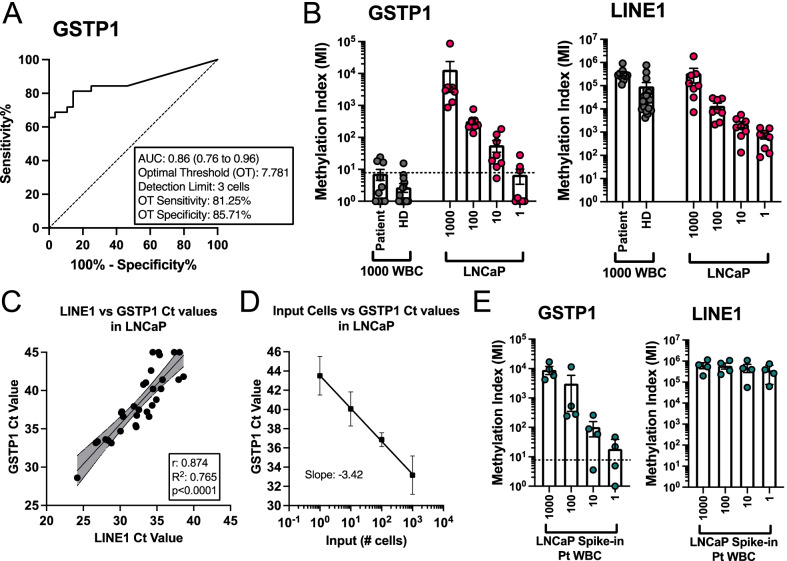
Range of detection of *GSTP1* promoter in DNA enriched by SEEMLIS. Methylated DNA was enriched by MBD2-MBD capture from DNA extracted from serially diluted LNCaP cells (*n* = 8 per dilution) and 1000 patient-derived (*n* = 10) or healthy donor (HD) (*n* = 18) white blood cells (WBCs). Quantitative PCR for *GSTP1* and *LINE1* was performed using enriched methylated DNA. **A** An ROC curve for all WBC samples and all LNCaP samples was created. Area under the curve (AUC) with 95% confidence interval is indicated. Optimal threshold (OT) values determined by Youden’s J statistic are listed with their associated sensitivity and specificity values. Detection limit was calculated using the slope of the best fit line of *GSTP1* Ct values plotted against cell input. **B** MI was calculated by delta Ct relative to a max cycle value (MCV) of 45 with all undetected samples set to a Ct value of 45 for analysis. Optimal threshold as determined by ROC curve is shown as a dotted line. Each dot represents an individual sample taken from a pool of cells diluted to the indicated concentration. **C** *LINE1* and *GSTP1* Ct values were plotted against each other for all LNCaP samples. A simple linear regression was performed to determine the best fit line and 95% confidence interval for that line (shaded region). *R* and *R*^2^ values are listed for the correlation. **D** *GSTP1* Ct values were averaged for each cell input and plotted against the cell input values. A semi-log nonlinear fit was performed to determine the best fit line and the slope of the best of fit line (− 3.42). Each tenfold dilution of input should result in a gain of 3.32 Ct values, giving a slope of − 3.32 for a perfect assay (100% efficiency). **E** MI for serially diluted LNCaP cells spiked into 1000 WBCs from a patient (*n* = 8) is shown. Performed as described above for **B**. All error bars represent standard error of the mean (SEM)

To determine the sensitivity, specificity, and range of SEEMLIS for *GSTP1* in LNCaP cells, we generated serial dilutions of LNCaP DNA at 1000, 100, 10, and 1 cell(s) and used 1000 WBCs as a negative control. Enrichment was performed as described in the methods and Fig. 1. DNA was digested with AluI and HhaI restriction enzymes prior to enrichment of methylated DNA with MBD2-MBD-coated magnetic beads. Quantitative PCR (qPCR) was performed on enriched DNA using primers for *GSTP1* and *LINE1*. Raw Ct values were used to create ROC curves and calculate *GSTP1* and *LINE1* methylation index (MI) for each dilution of LNCaP cells and WBC samples. An ROC curve was created using all LNCaP and WBC values, which had an area under the curve (AUC) of 0.86 (Fig. 2A). Youden’s J statistic was used to find the optimal threshold (OT), which resulted in 81.25% sensitivity and 85.71% specificity for methylated *GSTP1* to distinguish LNCaP from WBC. This threshold was applied to the methylation index values calculated for each dilution of LNCaP and WBC samples (Fig. 2B). We were able to detect methylated *GSTP1* from LNCaP cells above the OT 8/8 times at the 1000 and 100 cell dilutions, 7/8 times at the 10 cell dilution, and 3/8 times at the single cell dilution. GSPT1 was found above the OT 4/28 times in any WBC sample. *LINE1* methylation was detected in all samples in an input-dependent manner, indicating successful enrichment of methylated DNA from all sample types (Fig. 2B).

We also created an ROC curve using only LNCaP samples with greater than or equal to 10 cells to demonstrate the increased sensitivity when not working with single cell samples (Additional file 1: Figure S2). This curve results in an AUC very close to 1.0 (0.98). A similar threshold under these conditions results in an improvement in sensitivity (95.83%), but no improvement to specificity. The threshold determined to be optimal by Youden’s J statistic for this sample set is more restrictive, but with higher sensitivity and specificity of 87.50% and 96.43%, respectively. The ROC curve generated with data from LNCaP cell inputs of greater than or equal to 100 cells has an AUC of 1.0 with 100% sensitivity and specificity at the calculated optimal threshold (Additional file 1: Figure S2).

To determine the efficiency of SEEMLIS and calculate a detection limit, we plotted the *GSTP1* Ct values against *LINE1* Ct values and cell input number. *GSTP1* and *LINE1* Ct values were positively correlated to each other across the various dilutions of LNCaP cells (Fig. 2C). Similar relative enrichment of *GSTP1* and *LINE1* in individual enrichment reactions suggests stochastic variations in how much input DNA is added from the serial dilutions is responsible for differences in *GSTP1* enrichment from the same dilution, rather than inefficient capture of methylated DNA. Importantly, *GSTP1* is detected in MBD2-MBD-enriched LNCaP DNA in an input-dependent manner, where each ten-fold dilution of starting cells results in a gain of 3.42 Ct values as determined by the slope of the best fit line when plotting raw Ct values vs. log cell input, resulting in an assay efficiency of 96.06% (Fig. 2D). The equation of the best fit line was used to calculate a detection limit for *GSTP1* in this assay based on the OT from Fig. 2B. The detection limit based on these values is 3 cells.

### Range of detection of GSTP1 promoter in heterogenous samples after methylation-sensitive restriction enzyme digestion and MBD2-MBD enrichment

Next, we wanted to determine range of detection of *GSTP1* in samples representative of the purity of DNA isolated from circulating tumor cells (CTCs). DNA collected from CTC samples is often contaminated with large amounts of WBC DNA due to persistence of WBCs in the sample even after CTC enrichment. To test the ability of SEEMLIS to detect *GSTP1* in impure, heterogenous populations, we generated contrived samples to be representative of the purity levels we may obtain from CTC samples. We used LNCaP cells to represent CTCs and spiked them into patient-derived WBCs. We spiked approximately 1000, 100, 10, and 1 LNCaP cells by serial dilution into 1000 WBCs. These spike-ins represent a range of purity levels from CTCs being 50% of the population to “worst-case scenario” purity levels, where CTCs are less than 1% of the cell population. The input amounts are representative of the range of CTC numbers we may get from patient samples, although we also observe higher numbers of CTCs and greater purity in certain patients. We were able to detect methylated *GSTP1* from the contrived sample sets at similar levels as LNCaP cells alone at each dilution (Fig. 2E). Methylated *GSTP1* was detected from LNCaP cells spiked into 1000 WBCs above the OT 4/4 times at the 1000 LNCaP cell dilution, 3/4 times at the 10 LNCaP cell dilution and 2/4 times at the single LNCaP cell dilution. Detection of *LINE1* methylation corresponded to the total cell number present in the sample for each of these spike-in experiments.

We also wanted to confirm that different levels of WBCs would not introduce more false-positive *GSTP1* signals compared to 1000 WBC inputs, since circulating tumor cell samples may have varying levels of WBCs. We measured *GSTP1* and *LINE1* detection in WBC inputs of 5000, 2000, 100, 10, and 1 cells to confirm that *GSTP1* signal was consistently low in various WBC input amounts (Additional file 1: Figure S3). The *GSTP1* signal in WBCs did not increase significantly at inputs greater than 1000 cells. *GSPT1* signal was below the detection limit 100% of the time in dilutions lower than 1000 cells. *LINE1* was detected relative to input amount up to 2000 cells, where saturation of *LINE1* signal occurred. Therefore, in these assay conditions, interpretation of *LINE1* as a readout of total cellular level is only applicable to inputs of 2000 cells or less.

### Detection of methylated GSTP1 in prostate cancer CTCs

**Fig. 3:**
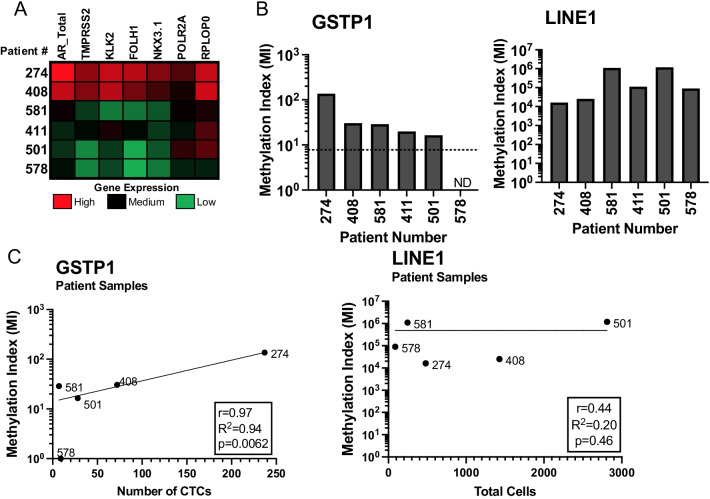
Detection of *GSTP1* promoter in SEEMLIS-enriched DNA from prostate cancer CTCs. Circulating tumor cells (CTCs) were enriched by positive selection for EpCAM using ESP. RNA and DNA were extracted from the EpCAM-selected population following live cell, on chip imaging of selected cells. **A** Gene expression was determined by qPCR for the indicated genes. Raw Ct values were used to create the heat map. Heat map intensity is determined separately for each gene, and comparisons can be made within each column, but not across rows. **B** DNA extracted from the enriched population was digested with AluI and HhaI restriction enzymes prior to enrichment of methylated DNA by MBD2-MBD. qPCR was performed for *GSTP1* and *LINE1* using the enriched methylated DNA. MI was calculated by delta Ct relative to a max cycle limit of 45 with all undetected samples (ND) set to a Ct value of 45 for analysis. Optimal threshold for *GSTP1* is shown as a dotted line. **C** MI for *GSTP1* and *LINE1* is plotted against the number of CTCs (*GSTP1*) or total number of cells (*LINE1*). A semi-log nonlinear fit was performed to determine the best fit line. *GSTP1* is positively correlated to CTC number with an *R*^2^ value of 0.94 and *p* value of 0.0062

A cohort of patients was selected to test the performance of SEEMLIS on patient CTC samples. The samples in this cohort were collected by EpCAM-positive selection following CD45+ depletion using a handheld ESP device called the VERSA where RNA was extracted prior to DNA extraction [35]. The VERSA technology integrates multiple analysis steps into one microfluidic device, including capture, staining, and imaging of CTCs followed by nucleic acid extraction from the same cells. The RNA from the samples in this cohort was used to assess gene expression of various prostate-specific markers to ensure the presence of CTCs in the sample. All samples had gene signatures that support the presence of prostate epithelial cells by expression of multiple prostate-specific genes (Fig. 3A). Estimates of CTC and WBC numbers and sample purity were made from images taken prior to nucleic acid extraction, where CTCs were considered EpCAM and Hoechst positive and negative for WBC exclusion markers CD45, CD14, and CD66b (Table 1). Sample 274 had a purity greater than 50%. Sample 411 did not have an image available. All other samples ranged in purity from 1.0 to 10.2%. CTC number ranged from 7 to 237.

**Table 1:** Characteristics of circulating tumor cell (CTC) samples

Patient #	CTCs	WBCs	Total cells	Purity (%)
274	237	242	479	49.5
408	72	1355	1427	5.0
581	7	238	245	2.9
411	NA	NA	NA	NA
501	28	2783	2811	1.0
578	9	79	88	10.2

Cell counts for CTCs, WBCs, and total cells are indicated. Purity percentage was calculated by dividing the number of CTCs by the total cell number. Patient 411 did not have an image available to generate cell counts

DNA obtained from the patient samples was enriched for methylated DNA by MBD2-MBD, and qPCR was performed for *GSTP1* and *LINE1* using the enriched DNA. Methylation index was calculated for each sample and the OT determined for *GSTP1* in LNCaP cells was applied. We were able to detect methylated *GSTP1* above the OT in 5/6 (83%) samples (Fig. 3B). *GSTP1* methylation index was significantly correlated to the CTC number determined by imaging (Fig. 3C). While *LINE1* methylation index did not significantly correlate to total cell number, *LINE1* methylation was successfully detected in all samples, including sample 578 where methylated *GSTP1* was not detected, suggesting *LINE1* methylation can be a useful control for capture of methylated DNA overall, even when target genes are not detected. Discrepancies in *LINE1* methylation and cell number may be due to inaccuracy in determining total cell number from live cell images or loss of *LINE1* methylation seen during prostate cancer progression leading to *LINE1* methylation levels not correlating with cell number [44]. This pilot study shows that SEEMLIS can successfully detect methylated *GSTP1* from a range of input target cell numbers and sample purities for seamless integration of targeted methylated DNA analysis into CTC capture, imaging, and gene expression analysis to allow comprehensive biomarker evaluation.

### SEEMLIS performance without the use of a methylation-sensitive enzyme to facilitate multiple target detection

While detection of one gene is useful in many contexts, there are myriad reasons for wanting to detect methylation in multiple gene targets from the same low-input sample. Splitting the sample into separate reactions for each gene is not ideal when working with heterogenous, low-input samples. Multiplexing primers in a pre-amplification reaction would allow analysis of multiple genes from the same limited sample, while still allowing for a single methylated DNA enrichment per sample. However, due to the complexity of methylation patterns in each different gene region that we may want to include, the use of a methylation-sensitive enzyme in the assay poses a problem.

A methylation-sensitive enzyme will cut at its restriction site only if the DNA is unmethylated. Designing primers that contain this restriction site can improve background from non-specifically captured DNA by preventing amplification during qPCR. However, in the context of evaluating methylation at multiple targets in low-input samples, this requires each restriction site for this enzyme to be methylated in each gene in the list of targets to be analyzed. While the use of the methylation-sensitive enzyme is beneficial for reducing the amount of background unmethylated target DNA, finding a methylation-sensitive restriction enzyme that is compatible with each region of interest becomes prohibitively difficult as the number of targets increases. Therefore, depending on the chosen genes, it may be necessary to use only non-methylation-sensitive restriction enzymes for detection in the same low-input sample.

We tested the effect of not using a methylation-sensitive enzyme on *GSTP1* detection in LNCaP and WBCs pre- and post-MBD2-MBD enrichment (Additional file 1: Figure S4). Samples were digested with the methylation-sensitive enzyme, HhaI or the non-methylation-sensitive enzyme, HpyCH4V. All samples were also digested with the non-methylation-sensitive enzyme AluI. We chose to add an additional non-methylation-sensitive enzyme to replace HhaI to increase the number of cut sites in our target regions. This ensures that the fragments are small enough to mitigate false positives from enrichment of methylated regions far away from the primer site. We found that while the use of the methylation-sensitive enzyme reduces signal from WBCs, the signal without the enzyme is still low enough to warrant use in heterogenous populations, if we apply a higher limit of detection to limit false positives. The combination of enzyme types should be empirically determined by the researcher for each application of this assay based on the desired target regions.

### Detection of multiple targets by pre-amplification of MBD2-MBD enriched DNA

In order to detect methylation at multiple genes from low cell input samples, we tested the addition of a pre-amplification step prior to qPCR. We used the TaqMan system for targeted pre-amplification, which amplifies pre-selected targets with the same probes used in subsequent qPCR-based analysis. We chose this targeted method for pre-amplification rather than a traditional whole-genome amplification (WGA) method for its speed, reduced hands-on time, and flexibility. Additionally, because we are analyzing a known subset of genes, amplifying the entire genome increases the risk of unpredictable biased amplification, which may be exacerbated by the uneven fragment lengths produced by restriction enzyme digestion. We have published multiple studies using the TaqMan system of pre-amplification for gene expression and have done extensive testing in this context to ensure pre-amplification is not affecting the results of our experiments [32, 35, 45]. In order to determine that this is true for our DNA probes, we compared Ct values for each primer using 5 ng and 0.5 ng of pre-amplified and unamplified LNCaP DNA (Additional file 1: Figure S5A). The amplification pattern is similar across all primer sets after pre-amplification and at different starting concentrations. *GSTP1* gained approximately 10 Ct values. *RASSF1*, *APC*, and *RARB* gained approximately 12 Ct values.

**Fig. 4:**
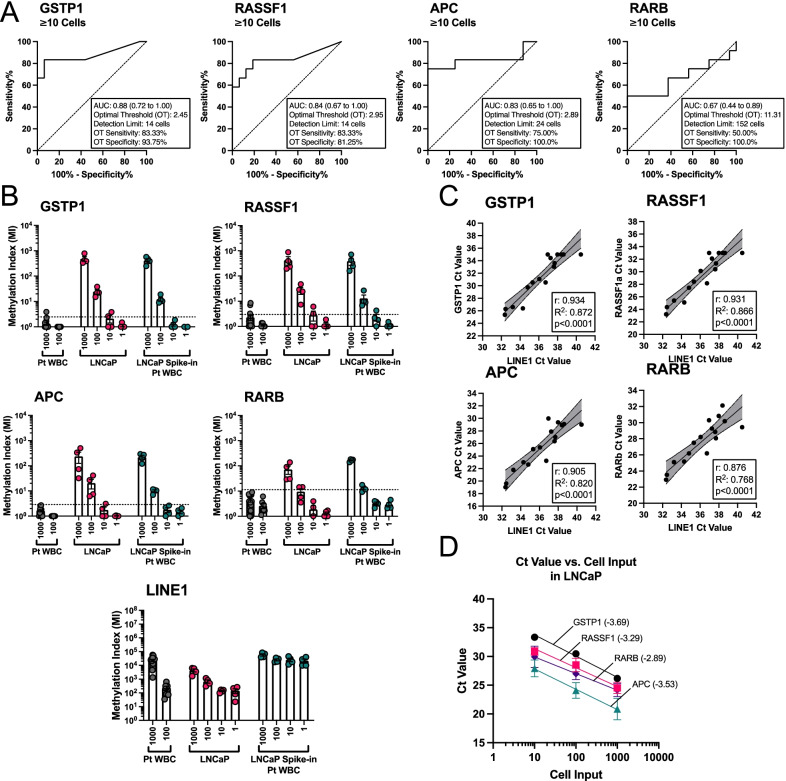
Detection of multiple genes from DNA enriched by SEEMLIS. Methylated DNA was enriched by MBD2-MBD capture from DNA extracted from serially diluted LNCaP cells (*n* = 4 per dilution), 1000 (*n* = 16) and 100 (*n* = 8) patient-derived WBCs, and serially diluted LNCaP cells spiked into 1000 patient-derived WBCs (*n* = 4 per dilution). Enriched methylated DNA was pre-amplified with probes to the indicated genes (excluding *LINE1*). Pre-amplified DNA was diluted 1:5 and qPCR was performed with the same probes, including *LINE1*. **A** For each gene, ROC curves for WBC samples of 1000 cells and LNCaP samples of 1000, 100, or 10 cells were created. Area under the curve (AUC) with 95% confidence interval is indicated. Optimal threshold (OT) values determined by Youden’s J statistic are listed with their associated sensitivity and specificity values. Detection limit was calculated using the slope of the best fit line of Ct values plotted against cell input **D**. **B** MI was calculated by delta Ct relative to a max cycles value (MCV) of 35 (*GSTP1*), 33 (*RASSF1*, *APC*, *RARB*), or 45 (*LINE1*) with all undetected samples set to the corresponding MCV for analysis. Optimal threshold as determined by ROC curve is shown as a dotted line. Each dot represents an individual sample taken from a pool of cells at the indicated concentration. **C** Ct values for *LINE1* versus Ct values for each gene were plotted against each other for LNCaP samples of 1000, 100, 10, and 1 cell(s). A simple linear regression was performed to determine the line of best fit and 95% confidence interval for that line (shaded region). *R* and *R*^2^ values are listed for the correlation. Each gene was significantly correlated to *LINE1* values with *p* values < 0.0001. **D** Ct values were averaged for each cell input and plotted against the cell input values. A semi-log nonlinear fit was performed to determine the line of best fit and the slope of the line of best fit, which are indicated in parentheses for each gene. All error bars represent standard error of the mean (SEM)

To test the performance of SEEMLIS with the pre-amplification step included, we generated serial dilutions of 1000, 100, 10, and 1 LNCaP cell(s) and used 1000 and 100 patient-derived WBCs as a negative control. We also spiked 1000, 100, 10, and 1 LNCaP cell(s) into 1000 patient-derived WBCs to mimic CTC samples. The samples were enriched for methylated DNA by MBD2-MBD capture. The enriched DNA was then placed directly into a pre-amplification reaction. We included primers for four genes in the pre-amplification pool (*RASSF1*, *APC*, *RARB* and *GSTP1*), which have previously been identified as being methylated in a large percentage of prostate cancers [19]. Primer locations, genomic context, and methylation level determined by whole genome bisulfite sequencing in LNCaP [41] and WBCs [42] are shown in Additional file 1: Figure S1. It is important to note that *RARB* is methylated at a low level in WBCs, which is likely to result in a more restrictive detection limit for this gene. *LINE1* was left out of the pre-amplification pool because it is abundant enough to be detected in samples that have been diluted after the pre-amplification without pre-amplification of *LINE1* itself. Pre-amplified DNA was then diluted 1:5 and qPCR was performed for *GSTP1*, *RASSF1*, *APC*, *RARB*, and *LINE1*. Ct values from the 1000 cell WBC samples and LNCaP samples of 10 cells or greater were used to create ROC curves for each gene (Fig. 3A). The 1 cell LNCaP dilution samples were not included in the ROC curves because of the increased background we expected from not using a methylation-sensitive enzyme for digestion. The AUC for *GSTP1*, *RASSF1*, *APC*, and *RARB* was 0.88, 0.84, 0.83, and 0.67, respectively. Youden’s J statistic was used to find the OT for each gene, which are listed in Fig. 4A.

Methylation index was calculated for each gene as described in the Methods section for pre-amplified samples and the OTs calculated for each gene in Fig. 4A were applied (Fig. 4B). We were able to detect methylated *GSTP1* and *RASSF1* from LNCaP cells above the OT 4/4 times at the 1000 and 100 cell dilutions and 2/4 times at the 10 cell dilution. We were able to detect methylated *APC* above the OT 4/4 times at the 1000 and 100 cell dilutions and 1/4 times at the 10 cell dilution. *RARB* was only detected above the optimal threshold 4/4 times at the 1000 cell dilution and 2/4 times at the 100 cell dilution. *GSTP1*, *RASSF1*, and *APC* were detected above the OT 4/4 times at the 1000 and 100 cell dilutions for the spike in samples. *RARB* was detected above the OT 4/4 times at the 1000 cell dilution and 3/4 times at the 100 cell dilution. Only *RASSF1* was ever detected above the optimal threshold at the 10 cell dilution for the spike-in samples (1/4 times). As expected, we were not able to detect any gene above the optimal thresholds at the 1 cell dilution for LNCaP alone or the spike-in samples. *LINE1* methylation was detected at all dilutions in all samples in a dilution dependent manner. *LINE1* Ct values were significantly positively correlated with Ct values for each gene (Fig. 4C). Methylated DNA was enriched in an input dependent manner for inputs of 1000, 100, and 10 cells for each gene, with efficiencies for *GSTP1*, *RASSF1*, *APC*, and *RARB* of 87%, 101%, 92%, and 122%, respectively (Fig. 4D). Undetected samples were excluded from efficiency analyses. Detection limits listed in Fig. 4A were calculated based on the OT using the equation of the line of best fit determined by plotting Ct values vs. cell input (Fig. 4D). *GSTP1* and *RASSF1* had a detection limit of 14 cells while *APC* and *RARB* had detection limits of 24 and 152 cells, respectively.

We also performed SEEMLIS on 100 WBCs in addition to 1000 WBCs to see whether lowering the amount of background cells would reduce background signal (Fig. 4B). The *GSTP1* and *RASSF1* signals from 100 WBCs were reduced to effectively 0. *APC* signal was reduced to the equivalent level of a single LNCaP cell. *RARB* signal was not reduced between 1000 and 100 WBCs. These results indicate that reducing the amount of background WBCs in the sample may lower the threshold enough to approach single cell sensitivity for certain genes.

### Detection of multiple targets in pre-amplified MBD2-MBD enriched DNA from cells isolated by single cell aspiration

**Fig. 5:**
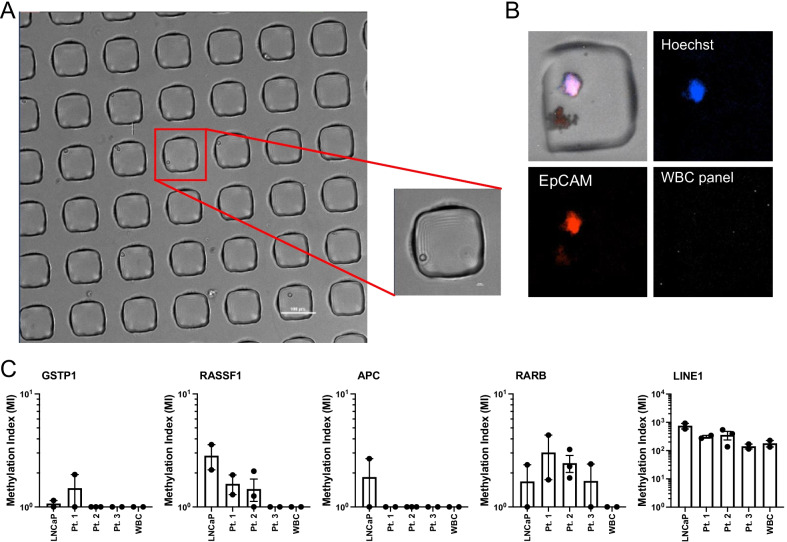
Detection of multiple genes in cells selected with single cell aspirator. **A** Brightfield image of single cell aspirator microwell array used to select individual LNCaP cells. **B** Representative image of a CTC in a microwell identified by immunofluorescent imaging as positive for Hoechst and anti-cytokeratin and negative for a panel of white blood cell antibodies. **C** Methylation index for indicated genes for groups of 10–15 CTCs aspirated from 3 patient samples. 10 LNCaP and 10 WBCs included as controls. All error bars represent standard error of the mean (SEM)

These data suggest that the lack of a methylation-sensitive enzyme and addition of a pre-amplification step can allow us to look at multiple gene targets in CTC samples where WBCs are present, but only in certain high-purity or high CTC-burden samples. In order to look at multiple gene targets in samples where purity or CTC-burden is low, further purification steps are warranted. One way to achieve 100% CTC purity is to use a single-cell aspirator to select only CTCs or only WBCs. We tested the feasibility of this by using a semi-automated single cell aspirator (SASCA) developed in our laboratory [46]. We tested the feasibility of this using 3 prostate cancer CTC samples that were first enriched for EpCAM-positive cells. We aspirated 2–3 groups of approximately 10–15 CTCs along with 10 LNCaP cells and WBCs as positive and negative controls, respectively. Figure 5A shows a representative single LNCaP cell in the microwell array that would be aspirated for analysis. A representative image of a selected CTC is shown in Fig. 5B. CTCs were identified as being Hoechst and EpCAM positive and negative for a panel of WBC markers: CD45, CD11b, CD34, and CD27. We then performed SEEMLIS without the use of a methylation-sensitive enzyme with the pre-amplification step included on each of these samples. Methylation index was calculated for each gene in each group (Fig. 5C). We were able to detect *LINE1* from each sample at comparable levels to each other and to our 10 cell dilution samples in Fig. 3. We were able to detect *GSTP1* from 1 LNCaP sample and in 1 CTC group from patient 1. *RASSF1* was detected in both LNCaP control groups as well as 2/2 CTC groups from patient 1 and 2/3 CTC groups from patient 2. *APC* was detected in 1/2 LNCaP groups, but was not detected in any CTC groups. *RARB* was detected in all CTC groups from patients 2 and 3 and in 1/2 CTC groups from patient 3. None of the genes, apart from *LINE1*, were detected in the aspirated WBC group. Generating pure samples by single cell aspiration allowed us to perform a multiplexed analysis of methylation signatures in prostate cancer CTCs. These data demonstrate the utility of this assay for targeted methylation analysis in CTCs and other low-input samples.

## Discussion

Analyzing epigenetic signatures in low-input samples such as DNA derived from liquid biopsies has been an ongoing challenge. Most techniques that measure DNA methylation rely on bisulfite conversion of DNA, which can lead to extensive damage of input material. This necessitates large input amounts, or reduces sensitivity such that targeted analysis of specific regions is not reliable. This is especially true in heterogenous samples due to the increased need for assay sensitivity to distinguish target DNA from background DNA. The assay presented here uses MBD2-MBD enrichment of methylated DNA in a semi-automated ESP-based system to support targeted DNA methylation analysis in low-input samples without chemical DNA alteration or lengthy protocols.

While we have developed SEEMLIS in the context of prostate cancer and specifically with CTC biomarker development in mind, this assay is readily transferable for targeted methylation analysis and biomarker development in any disease state, including blood and solid tumors and non-cancer diseases. This assay also provides a method for analysis of single cell heterogeneity, which may be useful not only for research in disease progression, but also for non-disease states such as development and cellular differentiation. In addition, adaptations to the SEEMLIS assay including the number and type of genes included in the analysis, the inclusion of more assay controls, and adjustments to patient selection criteria, such as setting a lower threshold of CTC burden, can readily be incorporated to improve clinical and disease-specific relevance. Future studies will be needed to identify gene sets, internal controls, and patient criteria that place the sensitivity and specificity of SEEMLIS into a clinically useful range.

SEEMLIS also lends itself to further downstream applications such as sequencing the MBD2-MBD-captured DNA (MBD-seq) and we are currently exploring this endpoint. There have been multiple studies that have performed reduced representation bisulfite sequencing (RRBS) from low-input samples including single cells, but this necessitates using sequencing methods that even further reduce sequence representation than when performed with thousands of cells [47, 48]. Furthermore, because the damage caused by bisulfite conversion is a random event, analysis of specific gene loci at the single cell level is reliant on chance that the site of interest remains intact, is converted efficiently, and is able to be mapped successfully post-sequencing. This assay provides a way to alleviate some of these challenges by enabling enrichment of methylated DNA from low-input samples that has not been chemically altered or damaged. While previous studies have demonstrated success in MBD-seq on samples with inputs as low as 5 ng [49], we anticipate that our semi-automated capture assay will be able to lower the minimum input even further based on the results in this study, especially in pure samples. As such, the clinical and research utility of this technique is far reaching.

A limitation of capture-based assays such as this one is the lack of single nucleotide resolution. As of now, bisulfite- or enzymatic-based conversion techniques must be used in order to achieve this level of resolution. However, resolution at the single base level is often not needed for clinical tests or to understand the contribution of DNA methylation to disease progression or heterogeneity. SEEMLIS is not meant to inform on the transcriptional nuances that may arise from alterations in methylation at specific CpG sites, but rather is designed to sensitively and specifically identify regions of DNA methylation in low-input samples. Identification of differences in methylated regions by this assay may then lead to more in-depth, higher-resolution studies of DNA from larger samples.

## Conclusions

This adaptability of SEEMLIS to many research-based and clinical needs, including highly sensitive single gene analysis and analysis of multiple genes from single or multiple cells, makes it a promising tool for methylation analysis in various settings. The ability to quickly and reliably analyze epigenetic biomarkers is important for researchers and clinicians due to revelations over the last few decades on epigenetic influence on development and disease.

## Materials and methods

### Cells for assay validation

LNCaP cells (ATCC) were a gift from Dr. David Jarrard and were cultured in RPMI medium (Corning) supplemented with 10% fetal bovine serum and 1% penicillin/streptomycin (HyClone). LNCaP cells were harvested when confluent and frozen in aliquots in growth medium plus 10% DMSO (Fisher Scientific) at -80 ºC and quickly thawed for use in assay validation experiments. White blood cells (WBCs) for assay validation experiments were derived from healthy donor blood or from the blood of a patient with prostate cancer. WBCs were selected on CD45 positivity using magnetic LS MACS columns (Miltenyi). WBCs were frozen in aliquots in PBS plus 10% DMSO (Fisher Scientific) at -80 ºC and quickly thawed for use in assay validation experiments.

### DNA extraction

Semi-automated DNA extraction was performed on a Gilson PIPETMAX liquid handling robot enabled for exclusion-based sample preparation (ESP), termed EXTRACTMAX [50]. LiDS buffer (90 mM Tris–HCL, 500 mM lithium chloride, 1% Igepal CA-630, 10 mM EDTA, 1 mM dithiothreitol) and MagneSil Paramagnetic Particles (PMPs) (Promega) resuspended in GTC buffer (10 mM Tris–HCl, 6 M guanidinium thiocyanate, 0.1% Igepal CA-630, pH 7.5) are added to the EXTRACTMAX extraction microplate (Gilson) by the robot. Cells were added to the microplate well containing LiDS, GTC, and MagneSil beads and mixed by the robot. Cells were allowed to lyse, and DNA was allowed to bind to MagneSil PMPs for 5 min. The robot then transferred the MagneSil PMPs with bound DNA by exclusion liquid repellency (ELR) through one PBST (PBS containing 0.1% Tween-20) wash, one PBS wash, and into water for elution. Beads were manually resuspended in the elution well and allowed to elute for 2 min. The MagneSil PMPs are magnetically transferred out of the elution well, leaving eluted DNA in water. LNCaP and WBC DNA for restriction enzyme and pre-amplification validation experiments were extracted using the AllPrep DNA/RNA mini kit (Qiagen) according to manufacturer’s instructions.

### Restriction enzyme digestion

DNA was digested using 1 µL of each chosen restriction enzyme (AluI at 10 units/µL, HhaI at 20 units/µL, and/or HpyCH4V at 5 units/µL; NEB) in 20 µL reactions containing 1 × CutSmart Buffer (NEB) for 15 min at 37 °C followed by enzyme inactivation for 20 min at 80 °C.

### Methylated DNA enrichment

25 µL of TALON magnetic beads (Takara) was washed 3 × with 100 µL 1 × Binding Buffer (BB) (4% glycerol, 1 mM MgCl_2_, 0.5 mM EDTA, 120 mM NaCl, 2 mM Tris–HCl pH 7.4, 0.2% Tween-20, and 0.5 mM DTT). Washed beads were resuspended in 100 µL MBD2-MBD Coupling Buffer (1 × BB, 1 × Halt protease inhibitor cocktail (Thermo Scientific), 500 ng Unmethylated Lambda DNA (Promega), 5 µL tagged MBD2-MBD (EpiXplore Kit, Takara) and placed on shaker at RT for 1 h to bind MBD2-MBD to the TALON beads. MBD2-MBD-bound beads were washed 3 × with 100 µL 1 × BB and resuspended in 88 µL 1 × BB with 1 × Halt protease inhibitor cocktail and added to 20 µL restriction enzyme-digested DNA in 200 µL PCR tubes. This reaction was placed on a shaker at RT for 3 h to bind methylated DNA to MBD2-MBD conjugated TALON beads. PCR tubes were placed onto the Gilson PIPETMAX liquid handling robot (EXTRACTMAN system enabled for ESP as previously described [50]) for washing and elution steps. The robot transferred the whole volume from the PCR tubes onto the EXTRACTMAX extraction microplate (Gilson) and then magnetically transferred the TALON beads through a wash containing 1 × BB with 1 × Halt protease inhibitor cocktail and into water for elution. The whole elution volume including beads was manually pipetted into new 200 µL PCR tubes and placed in a thermocycler at 95 °C for 15 min to ensure complete elution of methylated DNA. If pre-amplification of captured DNA was being performed, the elution volume was manually pipetted into new 200-µL PCR tubes containing the pre-amplification reaction mix and placed directly into the thermocycler under pre-amplification cycling conditions. The resulting eluate was used in downstream applications. Volumes indicated are per reaction.

### Quantitative real-time PCR and pre-amplification

Quantitative PCR was performed using TaqMan hydrolysis probes (Applied Biosystems) and iTaq Universal Probes Supermix (Bio-Rad). Custom primer pairs and FAM dye-labeled TaqMan MGB probes were used for methylation analysis. Primer and probe sequences are listed in Table 2. Commercially available probes from Applied Biosystems were used for gene expression analysis for *AR*_Total (Hs00907242_m1), *TMPRSS2* (Hs01120965_m1), *KLK2* (Hs_00428383_m1), *FOLH1* (Hs00379515_m1), *NKX3.1*(Hs00171834_m1), *POLR2A* (Hs00172187_m1), and *RPLP0* (4333761F). Cycling conditions: 5 min at 95 °C for initial denaturation and enzyme activation followed by 45 amplification cycles of 5 s at 95 °C and 30 s at 60 °C. Pre-amplification was performed using custom hydrolysis probes and TaqMan PreAmp Master Mix (Applied Biosystems) when indicated according to manufacturer specifications. Cycling conditions: 10 min at 95 °C for enzyme activation followed by 14 cycles of 95 °C for 15 s and 60 °C for 4 min. Pre-amplified samples were diluted 1:5 with TE buffer.

**Table 2:** Primer and probe sequences used in qPCR and pre-amplification of enriched methylated DNA

Gene	Primer	Sequence (5′–3′)
*GSTP1*	Forward	TTCGCTGCGCACACTTC
	Probe	CGGTCCTCTTCCTGCTGTCTGTTT
	Reverse	CTTTCCCTCTTTCCCAGGTC
*RASSF1*	Forward	CCTCCAGAAACACGGGTA
	Probe	TTTGCGGTCGCCGTCGTTGT
	Reverse	CTTCCTTCCCTCCTTCGTC
*APC*	Forward	TTATTACTCTCCCTCCCACCTC
	Probe	TCTTGTGCTAATCCTTCTGCCCTGC
	Reverse	TGGCAGTTGACACGCATAG
*RARB*	Forward	GAAGGAGAACTTGGGATCTT
	Probe	TTTCCAGGCTTGCTCGGCCAATC
	Reverse	AGCCTGTAATTGATCCAAATGA
*LINE1*	Forward	CGCAGGCCAGTGTGTGT
	Probe	CCGTGCGCAAGCCGA
	Reverse	TCCCAGGTGAGGCAATGC

Sequences for forward and reverse primers and internal hydrolysis probe are indicated for each gene. These sequences were used in the design and ordering of custom TaqMan probes for qPCR and pre-amplification of methylated DNA enriched by MBD2-MBD

### Single cell aspiration

For LNCaP and WBC aspiration, cells were first diluted to 100 cells/µL with PBS. For CTC aspiration, CTCs were first enriched using the VERSA platform as described above. The enriched cells were stained in the VERSA with Hoechst 33342 (Thermo Fisher) and antibodies to EpCAM conjugated to PE (Abcam) and exclusion markers: CD27, CD45, CD34, and CD11b each conjugated to AlexaFluor 647 (BioLegend). Single cell aspiration was performed using a custom *semi-automated single-cell aspirator* (SASCA) platform as previously described [46]. PDMS microarrays were prepared as previously described and adhered to a cleaned glass microscope slide. For diluted LNCaP and WBC samples, 6 µL cells were seeded for a total of ~ 600 cells per microarray. Stained CTC samples were seeded directly from the VERSA into the microarray. The microarray was imaged on a Nikon Ti-E Eclipse-inverted fluorescent microscope, and target cells were identified by phenotypic staining analysis for CTCs or brightfield imaging for LNCaP and WBC control groups. CTCs were identified as EpCAM-positive, exclusion (CD45/CD34/CD11b/CD27)-negative cells, whereas WBCs were classified as EpCAM-negative, exclusion-positive cells. Groups of 10–15 CTCs, LNCaPs, and WBCs were aspirated from microwells and dispensed directly into 10 µL PBS in the extraction plate for DNA extraction. Images of the microarray were analyzed using NIS Elements AR Microscope Imaging Software (NIS-Elements, RRID:SCR_014329).

### Whole blood processing and CTC capture

Blood was collected and processed as previously described [35, 51]. Briefly, whole blood collected by venipuncture into EDTA tubes was separated by centrifugation with Ficoll-Paque PLUS (Fisher Scientific). The layer containing mononucleated cells was depleted of CD45+ cells by magnetic LS MACS columns (Miltenyi). CTCs were isolated using an anti-EpCAM goat polyclonal antibody (R&D Systems). RNA was isolated using oligo (dT) Dynabeads (Invitrogen), and DNA was isolated using MagneSil Paramagnetic Particles (PMPs) (Promega) as previously described [34].

### Methylation index calculation

Methylation Index (MI) was calculated by the delta Ct method using the max cycle value (MCV) as the “control” Ct value:

$${\text{MI}} = { }2^{{ - \left( {{\text{Ct}} - {\text{MCV}}} \right)}} .$$

MCV for *GSTP1* (no pre-amplification) is 45. Unamplified wells were given the MCV as their Ct value for analysis. MI of 1 is then interpreted as no enrichment of methylated DNA for the target. MCV for each gene for pre-amplified samples was assigned for each gene by determining a Ct cutoff where serially diluted replicates are no longer reliably detected or where clustering of negative controls is seen (Additional file 1: Figure S5B). MCV for pre-amplified *GSTP1* was set at 35. MCV for pre-amplified *RASSF1* was set at 33, *APC at 28*, and *RARB* at 30. MCV for *LINE1* in either condition is 45. The MI calculation used here is based on the calculations used in the previously published method, COMPARE-MS [31].

### Statistical analysis

Receiver operator characteristic (ROC) curves were generated for each gene using Prism 8 (GraphPad) by plotting sensitivity vs. 100-specificity for the raw Ct values of LNCaP (true positive) and WBC (true negative). Optimal threshold (OT) values were determined using Youden’s J statistic, which is defined as the maximum value achieved from subtracting 100 from the sum of the sensitivity and 100-specificity values (in percentages). The associated Ct value was then converted into a methylation index (MI) using the MCV for each gene as described in the methods. Area under the curve (AUC) with 95% confidence intervals was found and reported, which indicate the probability that a randomly selected true-positive sample will have a greater MI value than a randomly selected true-negative sample. Simple linear regression and semi-log nonlinear fit analyses were performed in Prism 8 and r, R^2^ and slope are reported where relevant to data interpretation. Assay efficiencies (E) were calculated using slopes line of best fit when comparing raw Ct values to log cell input as follows:

$$E = - 1 + 10^{{ - \frac{1}{{{\text{slope}}}}}} .$$

All error bars represent SEM.

## Supplementary Information

**Additional file 1. Figure S1:** Primer locations and genomic context for GSTP1, RASSF1, APC, and RARB. **Figure S2:** ROC curves for GSTP1 detection from non-single cell sample groups. **Figure S3:** Detection of GSTP1 promoter in additional dilutions of WBCs. **Figure S4:** Effect of non-methylation-sensitive enzyme digestion on GSTP1 enrichment. **Figure S5:** Analysis of preamplification for GSTP1, RASSF1, APC, and RARB.

## Data Availability

Data for methylation levels in LNCaP and PBMCs and CpG island location are available on the GRCh37/hg19 genome assembly online at the UCSC Genome Browser.

## References

[1] Murrell A, Hurd PJ, Wood IC (2013). Epigenetic mechanisms in development and disease. Biochem Soc Trans.

[2] Greenberg MVC, Bourc'his D (2019). The diverse roles of DNA methylation in mammalian development and disease. Nat Rev Mol Cell Biol.

[3] Moore LD, Le T, Fan G (2013). DNA methylation and its basic function. Neuropsychopharmacol Off Publ Am Coll Neuropsychopharmacol.

[4] Deaton AM, Bird A (2011). CpG islands and the regulation of transcription. Genes Dev.

[5] Razin A, Riggs AD (1980). DNA methylation and gene function. Science (New York, NY).

[6] Ramchandani S, Bhattacharya SK, Cervoni N, Szyf M (1999). DNA methylation is a reversible biological signal. Proc Natl Acad Sci USA.

[7] Almouzni G, Cedar H (2016). Maintenance of epigenetic information. Cold Spring Harbor Perspect Biol..

[8] Strand SH, Orntoft TF, Sorensen KD (2014). Prognostic DNA methylation markers for prostate cancer. Int J Mol Sci.

[9] Yang M, Park JY (2012). DNA methylation in promoter region as biomarkers in prostate cancer. Methods Mol Biol (Clifton, NJ).

[10] Kobayashi Y, Absher DM, Gulzar ZG, Young SR, McKenney JK, Peehl DM (2011). DNA methylation profiling reveals novel biomarkers and important roles for DNA methyltransferases in prostate cancer. Genome Res.

[11] Ahmed H (2010). Promoter methylation in prostate cancer and its application for the early detection of prostate cancer using serum and urine samples. Biomark Cancer.

[12] Nakayama M, Gonzalgo ML, Yegnasubramanian S, Lin X, De Marzo AM, Nelson WG (2004). GSTP1 CpG island hypermethylation as a molecular biomarker for prostate cancer. J Cell Biochem.

[13] Zhao F, Olkhov-Mitsel E, van der Kwast T, Sykes J, Zdravic D, Venkateswaran V (2017). Urinary DNA methylation biomarkers for noninvasive prediction of aggressive disease in patients with prostate cancer on active surveillance. J Urol.

[14] Lawrence MS, Stojanov P, Mermel CH, Garraway LA, Golub TR, Meyerson M (2014). Discovery and saturation analysis of cancer genes across 21 tumor types. Nature.

[15] Fleischmann A, Saramaki OR, Zlobec I, Rotzer D, Genitsch V, Seiler R (2014). Prevalence and prognostic significance of TMPRSS2-ERG gene fusion in lymph node positive prostate cancers. Prostate.

[16] Pettersson A, Graff RE, Bauer SR, Pitt MJ, Lis RT, Stack EC, et al. The TMPRSS2:ERG rearrangement, ERG expression, and prostate cancer outcomes: a cohort study and meta-analysis. 2012. 10.1158/1055-9965.EPI-12-0042 PMC3671609 22736790

[17] Wang G, Zhao D, Spring DJ, DePinho RA (2018). Genetics and biology of prostate cancer. Genes Dev.

[18] Jernberg E, Bergh A, Wikstrom P (2017). Clinical relevance of androgen receptor alterations in prostate cancer. Endocr Connect.

[19] Yegnasubramanian S, Kowalski J, Gonzalgo ML, Zahurak M, Piantadosi S, Walsh PC (2004). Hypermethylation of CpG islands in primary and metastatic human prostate cancer. Cancer Res.

[20] Yegnasubramanian S, Wu Z, Haffner MC, Esopi D, Aryee MJ, Badrinath R (2011). Chromosome-wide mapping of DNA methylation patterns in normal and malignant prostate cells reveals pervasive methylation of gene-associated and conserved intergenic sequences. BMC Genomics.

[21] Majumdar S, Buckles E, Estrada J, Koochekpour S (2011). Aberrant DNA methylation and prostate cancer. Curr Genomics.

[22] Chimonidou M, Tzitzira A, Strati A, Sotiropoulou G, Sfikas C, Malamos N (2013). CST6 promoter methylation in circulating cell-free DNA of breast cancer patients. Clin Biochem.

[23] Chimonidou M, Strati A, Tzitzira A, Sotiropoulou G, Malamos N, Georgoulias V (2011). DNA methylation of tumor suppressor and metastasis suppressor genes in circulating tumor cells. Clin Chem.

[24] Kloten V, Becker B, Winner K, Schrauder MG, Fasching PA, Anzeneder T (2013). Promoter hypermethylation of the tumor-suppressor genes ITIH5, DKK3, and RASSF1A as novel biomarkers for blood-based breast cancer screening. Breast Cancer Res.

[25] Ellinger J, Haan K, Heukamp LC, Kahl P, Buttner R, Muller SC (2008). CpG island hypermethylation in cell-free serum DNA identifies patients with localized prostate cancer. Prostate.

[26] Darst RP, Pardo CE, Ai L, Brown KD, Kladde MP (2010). Bisulfite sequencing of DNA. Curr Protoc Mol Biol.

[27] Han D, Lu X, Shih AH, Nie J, You Q, Xu MM (2016). A highly sensitive and robust method for genome-wide 5hmC profiling of rare cell populations. Mol Cell.

[28] Taiwo O, Wilson GA, Morris T, Seisenberger S, Reik W, Pearce D (2012). Methylome analysis using MeDIP-seq with low DNA concentrations. Nat Protoc.

[29] Brinkman AB, Simmer F, Ma K, Kaan A, Zhu J, Stunnenberg HG (2010). Whole-genome DNA methylation profiling using MethylCap-seq. Methods (San Diego, CA).

[30] Sonnet M, Baer C, Rehli M, Weichenhan D, Plass C (2013). Enrichment of methylated DNA by methyl-CpG immunoprecipitation. Methods Mol Biol (Clifton, NJ).

[31] Yegnasubramanian S, Lin X, Haffner MC, DeMarzo AM, Nelson WG (2006). Combination of methylated-DNA precipitation and methylation-sensitive restriction enzymes (COMPARE-MS) for the rapid, sensitive and quantitative detection of DNA methylation. Nucleic Acids Res.

[32] Sperger JM, Strotman LN, Welsh A, Casavant BP, Chalmers Z, Horn S, et al. Integrated analysis of multiple biomarkers from circulating tumor cells enabled by exclusion-based analyte isolation. Clin Cancer Res Off J Am Assoc Cancer Res. 2016. 10.1158/1078-0432.CCR-16-1021 PMC5226928 27401243

[33] Casavant BP, Guckenberger DJ, Berry SM, Tokar JT, Lang JM, Beebe DJ (2013). The VerIFAST: an integrated method for cell isolation and extracellular/intracellular staining. Lab Chip.

[34] Strotman L, O'Connell R, Casavant BP, Berry SM, Sperger JM, Lang JM (2013). Selective nucleic acid removal via exclusion (SNARE): capturing mRNA and DNA from a single sample. Anal Chem.

[35] Pezzi HM, Guckenberger DJ, Schehr JL, Rothbauer J, Stahlfeld C, Singh A (2018). Versatile exclusion-based sample preparation platform for integrated rare cell isolation and analyte extraction. Lab Chip.

[36] Maldonado L, Brait M, Loyo M, Sullenberger L, Wang K, Peskoe SB (2014). GSTP1 promoter methylation is associated with recurrence in early stage prostate cancer. J Urol.

[37] Mahon KL, Qu W, Devaney J, Paul C, Castillo L, Wykes RJ (2014). Methylated glutathione S-transferase 1 (mGSTP1) is a potential plasma free DNA epigenetic marker of prognosis and response to chemotherapy in castrate-resistant prostate cancer. Br J Cancer.

[38] Yang AS, Estecio MR, Doshi K, Kondo Y, Tajara EH, Issa JP (2004). A simple method for estimating global DNA methylation using bisulfite PCR of repetitive DNA elements. Nucleic Acids Res.

[39] Cordaux R, Batzer MA (2009). The impact of retrotransposons on human genome evolution. Nat Rev Genet.

[40] Fu LJ, Ding YB, Wu LX, Wen CJ, Qu Q, Zhang X (2014). The effects of lycopene on the methylation of the GSTP1 promoter and global methylation in prostatic cancer cell lines PC3 and LNCaP. Int J Endocrinol..

[41] Pidsley R, Zotenko E, Peters TJ, Lawrence MG, Risbridger GP, Molloy P (2016). Critical evaluation of the Illumina MethylationEPIC BeadChip microarray for whole-genome DNA methylation profiling. Genome Biol.

[42] Li Y, Zhu J, Tian G, Li N, Li Q, Ye M (2010). The DNA methylome of human peripheral blood mononuclear cells. PLoS Biol.

[43] Song Q, Decato B, Hong EE, Zhou M, Fang F, Qu J (2013). A reference methylome database and analysis pipeline to facilitate integrative and comparative epigenomics. PLoS ONE.

[44] Yegnasubramanian S, Haffner MC, Zhang Y, Gurel B, Cornish TC, Wu Z (2008). DNA hypomethylation arises later in prostate cancer progression than CpG island hypermethylation and contributes to metastatic tumor heterogeneity. Cancer Res.

[45] Johnson BP, Vitek RA, Geiger PG, Huang W, Jarrard DF, Lang JM (2018). Vital ex vivo tissue labeling and pathology-guided micropunching to characterize cellular heterogeneity in the tissue microenvironment. Biotechniques.

[46] Tokar JJ, Stahlfeld CN, Sperger JM, Niles DJ, Beebe DJ, Lang JM (2020). Pairing microwell arrays with an affordable, semiautomated single-cell aspirator for the interrogation of circulating tumor cell heterogeneity. SLAS Technol.

[47] Guo H, Zhu P, Guo F, Li X, Wu X, Fan X (2015). Profiling DNA methylome landscapes of mammalian cells with single-cell reduced-representation bisulfite sequencing. Nat Protoc.

[48] Adey A, Shendure J (2012). Ultra-low-input, tagmentation-based whole-genome bisulfite sequencing. Genome Res.

[49] Aberg KA, Chan RF, Shabalin AA, Zhao M, Turecki G, Staunstrup NH (2017). A MBD-seq protocol for large-scale methylome-wide studies with (very) low amounts of DNA. Epigenetics.

[50] Guckenberger DJ, Pezzi HM, Regier MC, Berry SM, Fawcett K, Barrett K (2016). Magnetic system for automated manipulation of paramagnetic particles. Anal Chem.

[51] Schehr JL, Schultz ZD, Warrick JW, Guckenberger DJ, Pezzi HM, Sperger JM (2016). High specificity in circulating tumor cell identification is required for accurate evaluation of programmed death-ligand 1. PLoS ONE.

