# Supplementary material for: SEEMLIS: a flexible semi-automated method for enrichment of methylated DNA from low-input samples
Source: Clin Epigenetics. 2022 Mar 10;14:37. doi: 10.1186/s13148-022-01252-4 (PMC8908705; doi:10.1186/s13148-022-01252-4)
Supplement: Supplementary file 1 — Additional file 1. Figure S1: Primer locations and genomic context for GSTP1, RASSF1, APC, and RARB. Figure S2: ROC curves for GSTP1 detection from non-single cell sample groups. Figure S3: Detection of GSTP1 promoter in additional dilutions of WBCs. Figure S4: Effect of non-methylation-sensitive enzyme digestion on GSTP1 enrichment. Figure S5: Analysis of preamplification for GSTP1, RASSF1, APC, and RARB. [file 13148_2022_1252_MOESM1_ESM.docx]

**SUPPLEMENTARY FIGURES**

**Figure S1**

**Figure S2**

**Figure S3**

**Figure S4**

**Figure S5**

**SUPPLEMENTARY FIGURE LEGENDS**

**Figure S1: Primer locations and genomic context for *GSTP1*, *RASSF1*, *APC*, and *RARB*.** Forward and reverse primer locations are shown for each gene (red arrows). The boxes encompassing the red arrows represent the location on either side of the amplicon where the closest restriction enzyme cut site for any of the enzymes used in the assay will cut. Lightning bolts are locations of methylation-sensitive restriction enzyme cut sites in *GSTP1*. Genomic context for each gene was generated using the UCSC genome browser using the GRCh37/hg19 genome. The locations of the CpG island for each gene if present is indicated by the green bar with the total number of CpG dinucleotides in the whole CpG island (not only what is present in image) indicated. LNCaP (PMC5055731) and PBMC (PMC2976721) methylation data performed by whole genome bisulfite sequencing from studies deposited into the UCSC genome browser (PMC3855694) is shown by vertical gold bars.

**Figure S2: ROC curves for *GSTP1* detection from non-single cell sample groups.** ROCs curves for all WBC samples and LNCaP samples of 10, 100, and 1000 cells or 100 and 1000 cells were created. Area under the curve (AUC) with 95% confidence interval is indicated. Optimal threshold (OT) values determined by Youden’s J statistic are listed with their associated sensitivity and specificity values. Detection limits were calculating using the slope of the best fit line of *GSTP1* Ct values plotted against cell input.

**Figure S3: Detection of *GSTP1* promoter in additional dilutions of WBCs.** Additional dilutions of WBCs of 5000, 2000, 100, 10, and 1 cell(s) were created to determine the background *GSTP1* level and upper range of detection for the assay based on *LINE1* detection. *GSTP1* detection was not increased in larger dilutions compared to 1000 cells and was under the OT (dotted line) for all dilutions lower than 1000. *LINE1* values were dilution dependent for the 100, 10, and 1 cell(s) dilutions. The upper limit of range of detection is approximately 2000 cells.

**Figure S4: Effect of non-methylation-sensitive enzyme digestion on *GSTP1* enrichment.** 5 ng of LNCaP or WBC DNA was digested with AluI and either HhaI or HpyCH4V. Half of the samples were then enriched for methylated DNA. qPCR for *GSTP1* was performed on the digested and enriched DNA. Relative amplification of *GSTP1* is shown for each condition. Optimal threshold for *GSTP1* is shown as a dotted line.

**Figure S5: Analysis of pre-amplification for *GSTP1*, *RASSF1*, *APC*, and *RARB*. A)** 5 ng and 0.5 ng of LNCaP DNA was digested with AluI and HhaI. Digested DNA was then pre-amplified with primers to the indicated genes. qPCR was performed on pre-amplified (Pre-Amp) and non-pre-amplified (Input) DNA. Ct values are shown for each condition with standard deviation of triplicate qPCR wells shown. B) Ct values for SEEMLIS enriched methylated DNA from serial dilutions of LNCaP DNA and 1000 WBCs were plotted for each gene to determine max cycle value (MCV) cut off Ct value. Dotted lines indicate the determined cut off for each gene as labeled.
